# Monkeypox: a re-emergent virus with global health implications – a comprehensive review

**DOI:** 10.1186/s40794-024-00237-w

**Published:** 2025-01-15

**Authors:** Nourhan G. Naga, Enas A. Nawar, A’laa A. Mobarak, Aya G. Faramawy, Hend M. H. Al-Kordy

**Affiliations:** 1 Botany and Microbiology Department, Faculty of Science, Alexandria University, Alexandria, Egypt; 2 Department of Botany and Microbiology, Faculty of Science, Damanhour University, Damanhour, Egypt

**Keywords:** Monkeypox Virus (MPXV), *Orthopoxvirus*, Epidemiology, Diagnostic methods, Clade variability

## Abstract

Monkeypox virus (MPXV) is an enclosed, double-stranded DNA virus from the *Orthopoxvirus* genus, which also contains variola, vaccinia, and cowpox. MPXV, which was once confined to West and Central Africa, has recently had a rebound, spreading beyond its original range since 2017. The virus is distinguished by its unique morphology, which includes an oval or brick-shaped structure and a complex lipid and protein makeup. The current multi-country outbreak designated a public health emergency in 2022, has highlighted MPXV’s shifting epidemiology and ability to spread rapidly over the globe. ‘No one is safe until everyone is safe’ is a slogan we often heard during the COVID-19 pandemic, which is now also required for the growing global and regional mpox outbreaks. The epidemic is divided into two clades: Clade I and Clade II, which have distinct pathogenic characteristics. Diagnostic approaches have developed with advances in molecular techniques, yet problems persist in resource-constrained situations. This overview summarizes the virus’s history, epidemiology, morphology, and clinical characteristics, offering insights into its recent comeback and current global response efforts.

## Overview and background

Global health is under considerable risk from new and re-emerging infectious diseases, with zoonotic viruses posing a particularly dangerous threat to human populations. Current epidemics, such as those caused by zoonotic viruses like Coronavirus 2019 (COVID-19), Ebola virus, Dengue virus, Marburg virus, Avian influenza A virus (H5N1), Zika virus, and mpox (previously known as monkeypox), have highlighted the necessity of increased preparation and surveillance [1–6]. The Monkeypox virus (MPXV) is the causative agent of the zoonotic disease known as monkeypox (mpox). It is an enveloped virus with double-stranded DNA (dsDNA) and an oval or brick-shaped structure, belonging to the Chordopoxvirinae subfamily, the Poxviridae family’s, and *Orthopoxvirus* genus [7]. Other members of this genus include the Variola virus, Vaccinia virus, Cowpox virus, Taterapox virus, Camelpox virus, and Ectromelia virus. Throughout West and Central Africa, MPXV was endemic for fifty years [8], and it was rare for the virus to spread to non-endemic areas [9]. However, in a world that is becoming more interconnected, the recent mpox outbreak in 2022 has brought attention to the possible worldwide threat posed by zoonotic viruses. The outbreak quickly spread to other non-endemic nations. Since 2017, the prevalence of mpox has increased outside of endemic regions, and the disease’s epidemiological profile has changed within endemic areas [10]. As a result, in endemic countries in 2022, MPXV has emerged and reemerged [9]. Since monkeys are not the only animals that can spread the virus, the term “monkeypox” may be misleading. Squirrels, rats, mice, and other rodents are also known to transmit the virus. MPXV was first isolated from a species of monkey, *Macaca cynomolgus* (also known as *Macaca fascicularis*), when it was brought from Africa to Denmark. Later, in 1970, reports of the first MPXV case in humans came from Zaire, Central Africa [11].

### MPXV in past and present

Although the virus had previously affected apes and monkeys, there were no recorded cases of MPXV infection in humans before 1970 [12]. Monkey infections were first discovered in 1958 in captive monkeys in Denmark and were initially documented in laboratory and captive animals. In August 1970, a 9-month-old boy in the Democratic Republic of the Congo (DRC) became the first known human mpox case [13]. Subsequently, between September 1970 and April 1971, six more cases of mpox were identified in Sierra Leone, Nigeria, and Liberia [14]. Since then, reports of MPXV have come from several nations, and it is currently endemic in Benin., the Central African Republic, Cameroon, the DRC, Ivory Coast, Nigeria, Gabon, Liberia, South Sudan, the Republic of the Congo, and Sierra Leone [15]. In May 2022, the current worldwide mpox outbreak began [7, 16], and on July 23, 2022, it was declared a public health emergency of global importance [17]. By August 2023, 88,600 laboratory-confirmed cases and 152 deaths (case-fatality rate: 0.17%) had been recorded across 113 countries, 106 of which had not previously reported mpox cases. In 2022, during the mpox outbreak, significantly affected countries include Canada (*n* = 1,459), Mexico (*n* = 3,455), Peru (*n* = 3,561), Germany (*n* = 3,673), the United Kingdom (*n* = 3,730), Colombia (*n* = 3,880), France (*n* = 4,110) and Spain (*n* = 7,408). The Americas reported the highest number of cases, with Brazil (*n* = 10,168) and the USA (*n* = 29,513) accounting for 48.32% of all cases [18]. With 634 cases reported, Nigeria has the highest number of mpox cases in Africa.

### MPXV’s morphology and genomic organization

**Fig. 1:**
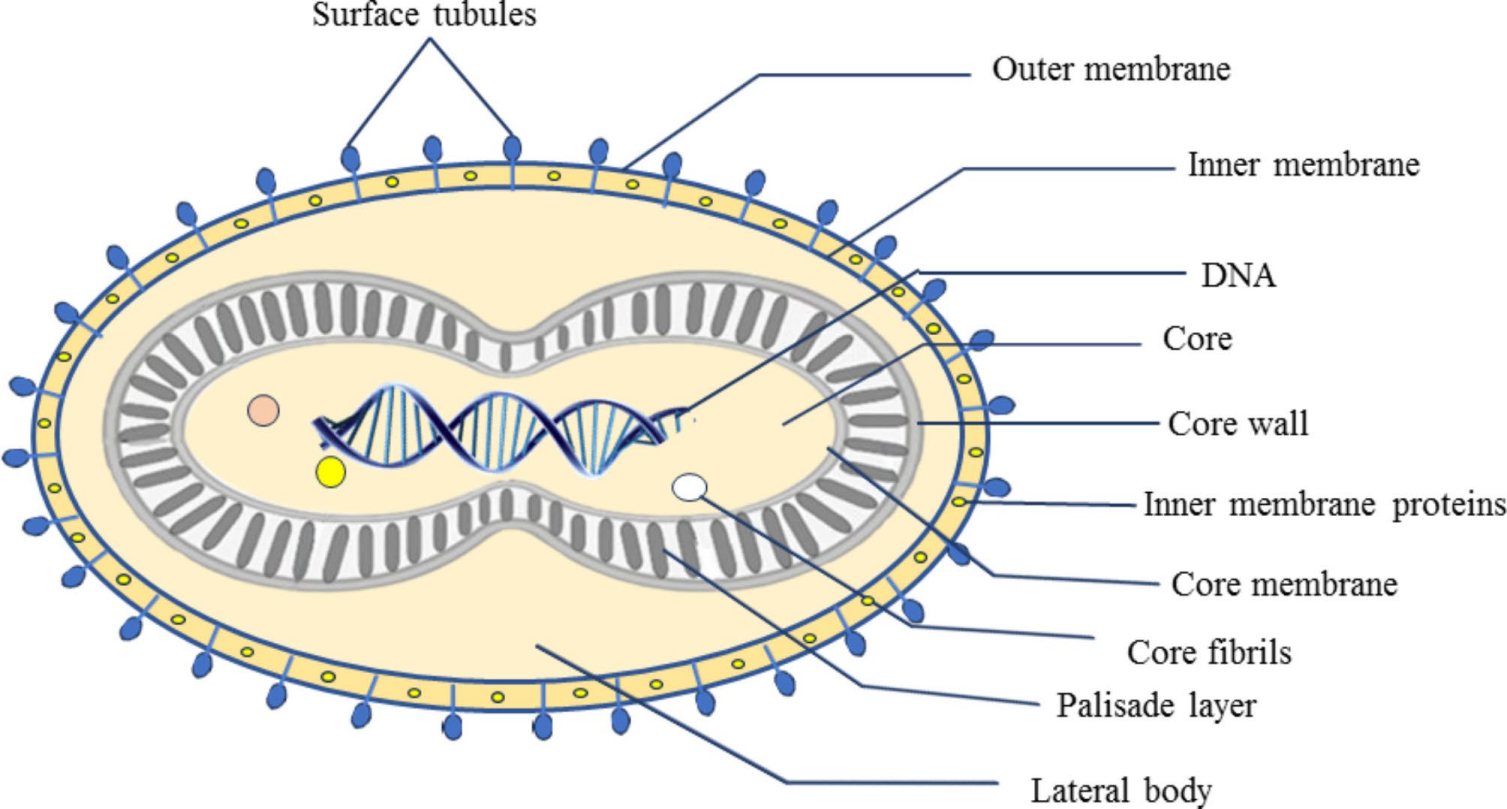
Structure of monkeypox virus (MPXV)

The MPXV virus is built architecturally, with ovoid or brick-shaped particles surrounded by lipoprotein transmembrane domains., similar to other orthopoxviruses. The size of MPXV is approximately 200 × 250 nm [19], making it visible under a light microscope, though ultra-resolution requires electron microscopy for detailed observation. The MPXV virion is composed of four main parts: the outer lipoprotein envelope, central core, outer membrane, and lateral bodies. The outer membrane, which features many tubules on the surface, encloses the palisade layer, lateral bodies, and the core [20]. The central core includes double-stranded DNA (dsDNA) from viruses and core fibrils, encircled by the palisade layer’s tightly packed rod-shaped structure **(**Fig. 1**)**. When viruses are released spontaneously, they typically retain the outer lipoprotein envelope, whereas viruses released through cellular rupture do not. The extracellular enveloped virus and the intracellular mature virus are the two infectious viral particles that are produced during replication [21]. The peripheral bodies and viral core, which include specific proteins, are encased within the lipoprotein envelope covering the intracellular mature virus’s surface [22]. A mature virion consists of about 80 viral proteins [23].

MPXV’s genome is a linear double-stranded DNA (dsDNA) molecule that is approximately 197 kilobase pairs (kbp) long and encodes over 190 open reading frames. These ORFs code for proteins important in viral replication, host immune evasion, and virion structure. The central portion of the genome is conserved among *Orthopoxviruses* and encodes proteins required for viral replication, such as DNA polymerases, helicases, and transcription factors. In contrast, the terminal sections of the genome are more changeable, and encode proteins that affect the host’s immunological response, such as complement-binding proteins, interleukin-1 beta-binding proteins, and apoptosis inhibitors [24, 25]. Furthermore, the MPXV genome contains multiple virulence factors, including proteins that limit host immune responses and promote viral survival in the host. For example, the MPXV encodes many ankyrin repeat proteins, which are believed to influence the host’s inflammatory response. MPXV’s genetic architecture enables it to adapt to many hosts and conditions, making it a potent zoonotic pathogen [26].

## Current status and spread

Following the 2018 outbreak in Nigeria, human monkeypox was identified in individuals who were visiting Nigeria or had come into contact with people outside of Africa who had been diagnosed with the monkeypox virus. Since May 14, 2022, several countries have reported clusters of cases of monkeypox; These infections have an unclear origin and there are no direct travel-related exposure risks [27]. The virus is divided into 2 clades: Clade I, which is also referred to as the Central African or Congo Basin clade, and Clade II, which is also called the West African clade. Clade I is split more into lineages 1–5 and Clade II into subclades IIa and IIb. Subclade IIb, which emerged in Nigeria, is currently responsible for the ongoing multi-country outbreak between 2022 and 2024 [28–30]. Pathogenicity, rates of transmission, and host immune response modification vary among these clades [31]. According to Sukhdeo [32], a human outbreak in the United States in 2003 was connected to the West African lineage, which is known to cause moderate diseases that have little transmission from one person to another. The Congo Basin clade, on the other hand, was discovered in humans for the first time in 1970 and is linked to increased morbidity and increased transmission between individuals (Table 1).

**Table 1:** Overview of MPXV Epidemiology

Year/Date	Location/Region	Clade/Lineage	Key Events	References
**2018**	Nigeria	West African clade	Human monkeypox identified in individuals visiting or in contact with travelers from Nigeria.	[25]
**May 14**,** 2022**	Multiple countries	Unclear origin	Clusters of mpox cases reported, no direct travel-related exposure risks.	[25]
**May 18–20**,** 2022**	Portugal, Spain, Canada, Belgium, Sweden, Italy, Australia	Clade IIb	First reported cases in several European countries and Australia during the multi-country outbreak.	[30, 32]
**May 29**,** 2022**	Global	Various clades	WHO raised a mild danger for global public health from mpox in non-endemic countries.	[32]
**July 23**,** 2022**	Global	Various clades	WHO declared the ongoing global monkeypox outbreak a public health emergency of international concern (PHEIC).	[32]
**November 4**,** 2022**	United States and globally	Various clades	CDC reported 78,229 confirmed cases and 41 deaths in 109 countries.	[33]
**April 11–13**,** 2024**	Kinshasa, Central and West Africa	Clade Ib (DRC), Clade IIa	High-level emergency meeting held to address the persistent mpox pandemic.	[27, 34]
**August 19**,** 2024**	Africa (multiple countries)	Clade Ib, Clade Ia, Clade II	WHO reported over 1,854 confirmed mpox cases in the WHO African Region, with a surge in the DRC.	[35]
**July 2024**	DRC, Burundi, Kenya, Rwanda, Uganda	Clade Ib	Emergence of MPXV clade Ib in new regions, associated with sexual contact and commercial sex networks.	[35]
**2024**	Republic of Congo, Central African Republic, Cameroon, Côte d’Ivoire, Liberia, Nigeria, South Africa	Clades Ia, IIa, IIb	Continued spread of MPXV clades across Africa, significant outbreaks affecting adults.	[35]

Genes determining virulence in the two monkeypox clades—West African and Central African—have been identified through genomic analysis. The West African clade’s open reading frames exhibit fragmentations and deletions, reducing its pathogenicity compared to the Central African clade [33].

Notably, on May 18, 2022, Portugal, Spain, and Canada reported 14, 7, and 13 cases of mpox infection, respectively. The following day, May 19, 2022, several other European countries, including Belgium, Sweden, and Italy, announced their first mpox cases. On May 20, 2022, two cases were also reported in Australia [32, 34]. On May 29, 2022, according to the World Health Organization (WHO), there was a mild danger for global public health from the country monkeypox epidemic among non-endemic countries, but since then, the UK has classified monkeypox as an infectious disease with severe repercussions.

On July 23, 2022, the WHO declared the ongoing global monkeypox outbreak a public health emergency of international concern. The WHO decided to take the unprecedented step of proclaiming a worldwide emergency for the second time in two years [34]. As of November 4, 2022, the Centers for Disease Control and Prevention (CDC) reported 78,229 confirmed cases of monkeypox and 41 fatalities across 109 nations. According to WHO data, the United States leads the list of the top 10 nations with the highest number of confirmed cases, followed by Brazil, Spain, France, the UK, Germany, Colombia, Peru, Mexico, and Canada. These countries together account for 86.4% of all recorded cases worldwide [35] (Table 1).

According to Satapathy [36], Pakistan has announced the first-ever fatality from monkeypox. The victim, a male in his forties had just gotten back from an excursion to Saudi Arabia additionally being HIV positive. He died from complications related to mpox. A doctor noted that all individuals infected with mpox in Pakistan had traveled from overseas, with no locally transmitted cases recorded. Visitors traveling from Middle Eastern countries have been associated with every case that has been found so far. Cevik [29] reported that from April 11 to 13, 2024, Kinshasa hosted a high-level emergency meeting on mpox. Participants agreed that multiple countries in Central and West Africa are facing an alarmingly persistent mpox pandemic. The major result of the conference was the agreement to create an international monkeypox treatment strategy for Africa, in response to calls for prompt and coordinated action after over fifty years of mpox outbreaks in the area. However, the findings of this a high degree conference were far less than anticipated. In addition to the effects of the disease on health and its economic and social consequences, there are also grave worries over the changing patterns of spread additionally the elevated death rate, particularly in kids. African nations, governments, and scientists must take immediate action to address these unmet needs, given the potential risk of the disease spreading to neighboring nations and abroad (Table 1).

According to the WHO, a meeting took place on August 19, 2024, to address the ongoing monkeypox epidemic [37] (https://www.who.int/news/item/19-08-2024-first-meeting-of-the-international-health-regulations-(2005)-emergency-committee regarding-the-upsurge-of-mpox-2024 ). In a summary of the global epidemiological situation regarding mpox, the WHO Secretariat noted that, as of the first half of 2024, the 1,854 confirmed mpox cases reported by States Parties in the WHO African Region accounted for 36% (1,854/5,199) of all cases observed globally. Of these, 95% (1,754/1,854) occurred in the DRC, which is currently experiencing a significant surge in mpox cases. Over 15,000 clinically compatible cases and more than 500 deaths have been reported, surpassing the DRC’s case count from 2023. Following WHO guidelines, MPXV clade Ib is a newly identified strain of the virus that first emerged in the DRC. It has been spreading eastward via transmission from person to person, most likely primarily through sexual relations. Although estimates suggest it appeared in September 2023, it was first described in 2024. The outbreak of clade Ib in the DRC predominantly affects adults and is escalating rapidly. It is primarily, though not exclusively, transmitted through sexual contact and is notably prevalent within networks associated with commercial sex and sex workers. Since July 2024, mpox cases related to MPXV clade Ib have been reported in four neighboring countries that had not previously documented mpox: Burundi, Kenya, Rwanda, and Uganda. These cases are epidemiologically and phylogenetically connected to the outbreak in the eastern provinces of the DRC. Additionally, mpox cases associated with MPXV clade Ia have been documented in the Republic of Congo and the Central African Republic in 2024, while instances related to MPXV clade II have been reported in Cameroon, Côte d’Ivoire, Liberia, Nigeria, and South Africa (Table 1).

The Committee received a briefing from representatives of Burundi, the DRC, Kenya, Rwanda, South Africa, and Uganda on the status of the mpox epidemic in their respective countries, including the needs, challenges, and ongoing response efforts. While reports of MPXV clade Ib-related mpox were generally limited, Burundi reported 100 confirmed cases of clade Ib-related mpox since July 2024. These cases were distributed across several districts, with children under five accounting for 28% of the reported cases. Additionally, the WHO Secretariat provided an overview of the measures already implemented to support preparedness and response efforts in States Parties at risk and experiencing a rise in mpox cases. These measures include: allocating USD 1.45 million from the WHO Contingency Fund for Emergencies; initiating the process of including two mpox vaccines on the Emergency Use Listing; collaborating with partners and stakeholders to ensure equitable access to vaccines, treatments, and diagnostics; and developing a regional response plan, which is projected to initially cost USD 15 million, among other actions [37] (Table 1).

### Transmission and risk factors

Monkeypox (mpox) is a zoonotic viral disease that can be transmitted in two different main routes: animal-to-human transmission and human-to-human transmission.

#### Animal-to-human transmission

**Fig. 2:**
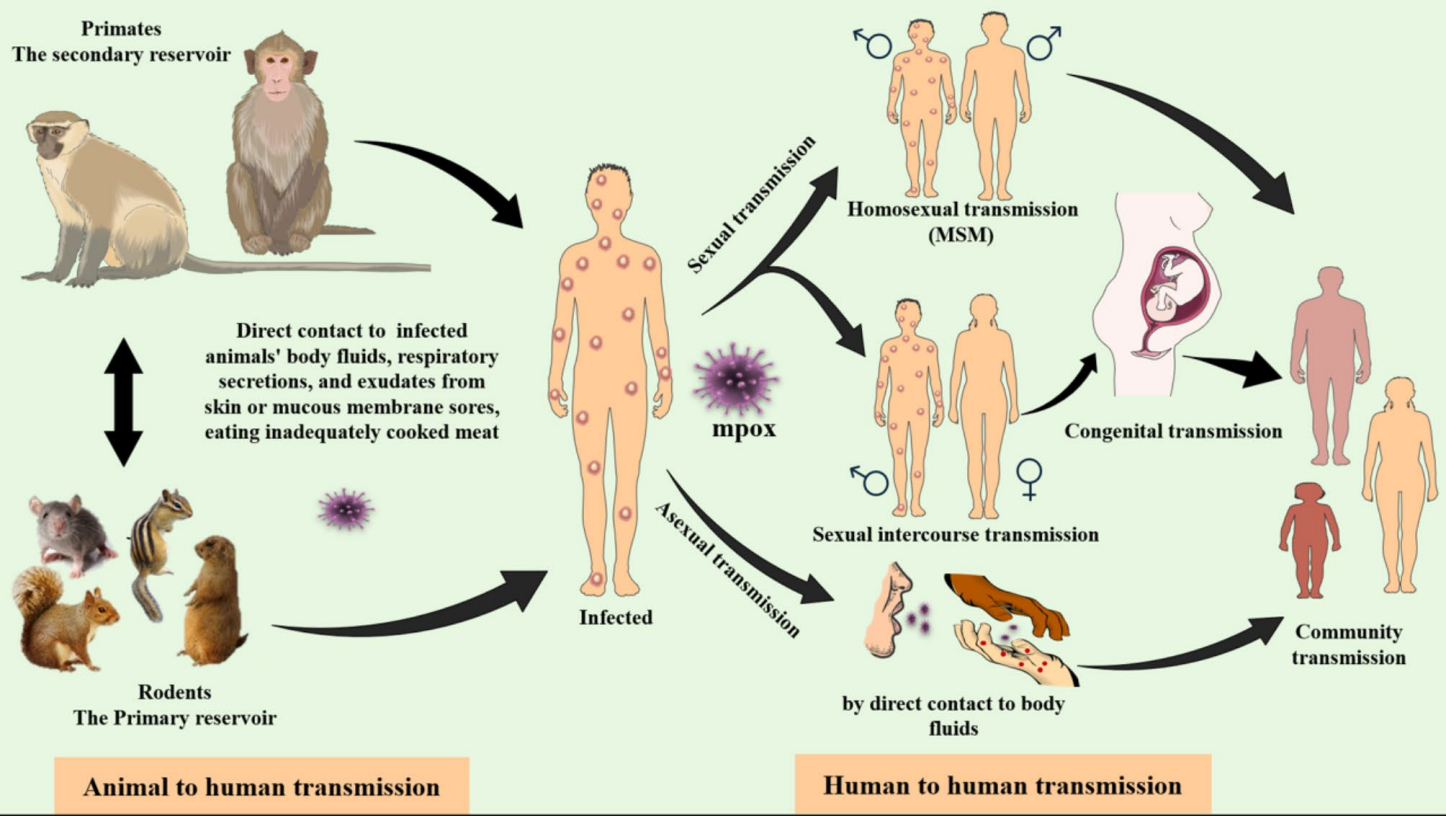
Schematic diagram showing different two ways of mpox transmission

The precise host reservoir for the mpox is still ambiguous. However, it is strongly believed that rodents or squirrels are the primary reservoirs for the mpox, which can be transmitted between a range of non-human primates including monkeys, prairie dogs, and anthropoid apes, through contact with respiratory droplets, skin lesions, or any bodily fluids. Animal-to-human transmission typically occurs via exposure to these infected animals’ body fluids, including saliva, respiratory secretions, and exudates from skin or mucous membrane sores, and through an animal bite. Additionally, virus-containing wastes such as the excretion of viral particles in feces and eating inadequately cooked meat from infected animals are significant sources for the transmission and spread of the virus (Fig. 2) [38–40]. The predominant regions impacted were the pastoral and rainforest areas of the Congo Basin, namely in the DRC. However, the infectivity has now disseminated, resulting in a surge in documented cases, particularly from the western and central regions of the African continent [40–42]. The risk factors related to zoonotic transmission include outdoor sleeping on the ground, residing close to the forest, where the infected animals are common, absence of smallpox vaccination, and contact with or consumption of deceased bushmeat or monkeys, are risk factors for the animal to human transmission of mpox [38, 42, 43].

#### Human-to-human transmission

Human-to-human transfer occurs less frequently than transmission from animal to human. Mpox is primarily transmitted between humans by extended physical encounters, inhaling large airborne respiratory droplets of the active virus, or direct contact with monkeypox eruptions, scabs, or body fluids. Physical contact with contaminated objects, textiles (clothes, bedding, or towels), and surfaces touched by an individual with mpox can further facilitate transmission [44, 45]. Although recent outbreaks, particularly during the 2022 epidemic, have revealed a high number of cases among males who engage in sexual activity with other males (MSM), mpox is primarily transmitted through close physical contact, including during sexual activity, and is not classified as a sexually transmitted infection. The virus has been identified in bodily fluids such as seminal fluid, but it spreads through skin-to-skin contact rather than only through sexual contact [41, 46–48]. Mpox can also transmitted through the placenta from mother to fetus (congenital monkeypox) [40, 43, 49]. Factors contributing to the probability of human-to-human transmission of mpox virus include sharing a room or bed, residing in a shared dwelling, and consuming food or beverages from a shared container [42, 50]. Also, people with diabetes mellitus, heart restrictions, or immunodeficiencies (HIV/AIDS) face a greater risk of severe mpox infection than healthy ones, or unvaccinated individuals against smallpox [51].

**Fig. 3:**
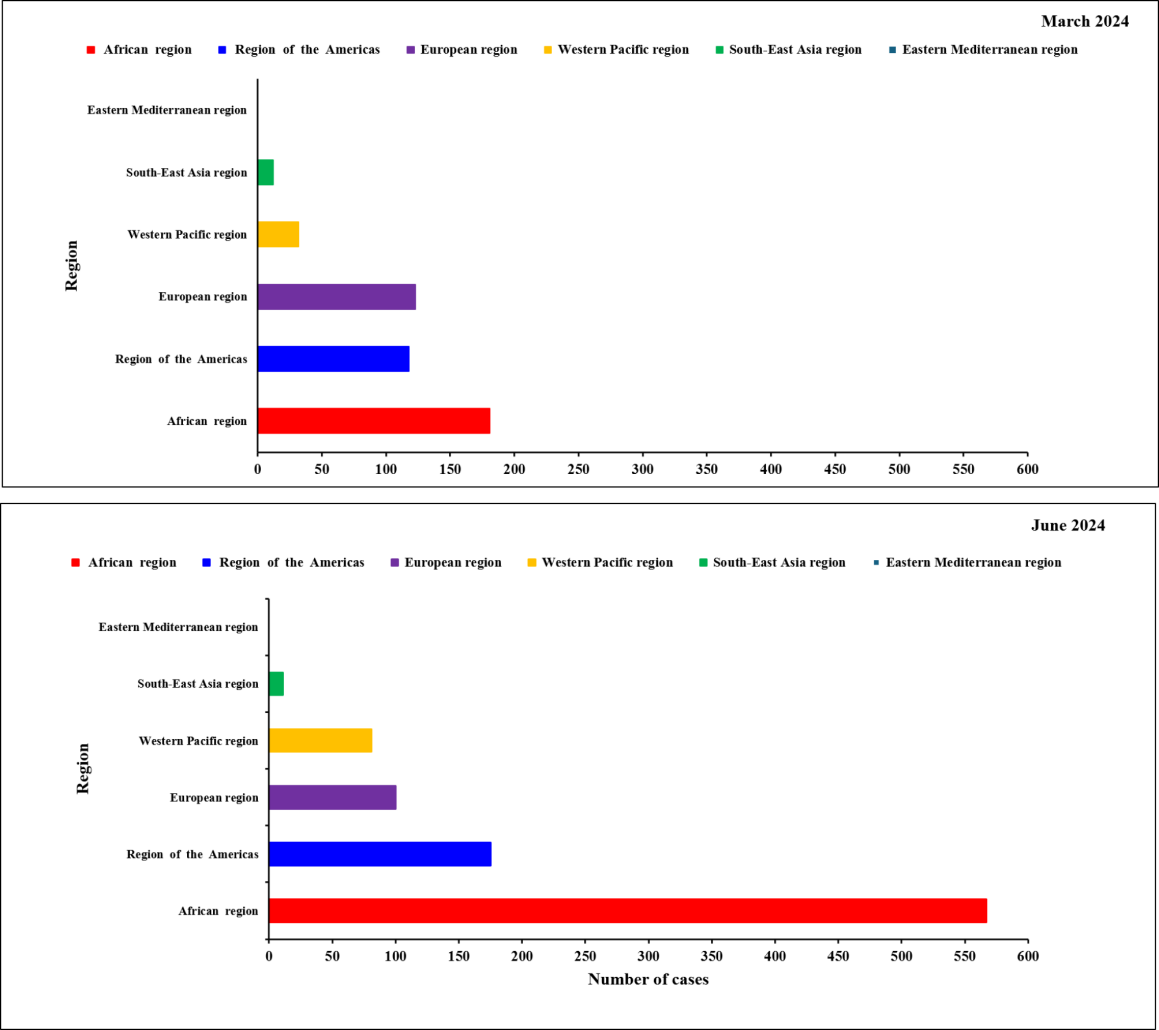
The increase rate of mpox transmission and number of cases through (March and June 2024)

On August 14, 2024, the WHO officially declared the mpox epidemic a global public health emergency. Comparing WHO reports data by March 2024 and June 2024, which reflect the continuing transmission of mpox and the sharp rise in cases number across the world especially in the African region which has the highest rate of mpox transmission, where the number of cases in March 2024 was (118 cases) while in June of the same year, the number of cases increased to (567 cases) (Fig. 3) [52].

## **Immune responses**, **symptoms**,** and severity**

*Orthopoxviruses*, including MPXV, have multiple tactics for evading the host’s immune system, aided by genomes that encode over 200 genes. These genes target different stages of the innate and adaptive immune responses, with some immunomodulatory proteins shared by the *Orthopoxvirus* family and others specific to MPXV. Immune evasion methods differ between MPXV clades, which contributes to pathogenicity differences. MPXV’s immune evasion techniques are divided into three stages: inhibiting innate immune identification, reducing inflammatory responses, and preventing adaptive immune activation. MPXV inhibits pathogen detection by the host’s pattern recognition receptors and reduces subsequent antiviral responses, such as type I interferon activation. Clade I MPXV boosts the AKT pathway, limiting apoptosis, while Clade II has mutations impacting IL-1β binding, reducing host control over the virus. Furthermore, MPXV disturbs T cell activation by competing with B7.1/B7.2 surface proteins, effectively inhibiting T cell signaling [47, 53, 54].

The clinical presentation of MPXV is consistent with its immune evasion strategies, as the virus manifests in symptoms that frequently mimic other poxviruses, including smallpox. The monkeypox virus is a member of the *Orthopoxvirus* genus. Its clinical manifestations are similar to those of smallpox, but generally milder [45]. As 90% of mpox patients present with enlarged lymph nodes, which are not commonly seen in smallpox, these swollen lymph nodes are considered a distinctive hallmark of mpox [55]. However, these lymph nodes could also be the result of the virus attempting to elude the host’s immunological response. The majority of mpox cases in humans present with mild to moderate symptoms and follow a self-limiting course. The disease’s manifestation can vary depending on factors such as the mode of transmission, host susceptibility, and the amount of virus introduced. Certain routes of exposure may lead to more severe illness, although these typically have shorter incubation periods [56]. Similar to smallpox, the infectious route of the MPXV starts with contact with the host’s oropharyngeal or respiratory mucosa. The virus first multiplies at the site of inoculation after entering the body. For human-to-human transmission, the respiratory and oropharyngeal mucosa are usually the sites of infection. The virus multiplies during initial viremia and then moves on to other lymph nodes. MPXV, which is well known for manipulating the immune system, also takes advantage of this period of viremia to propagate farther within the host. The viral burden spreads throughout the bloodstream to distant lymph nodes and organs in secondary viremia [38].

**Fig. 4:**
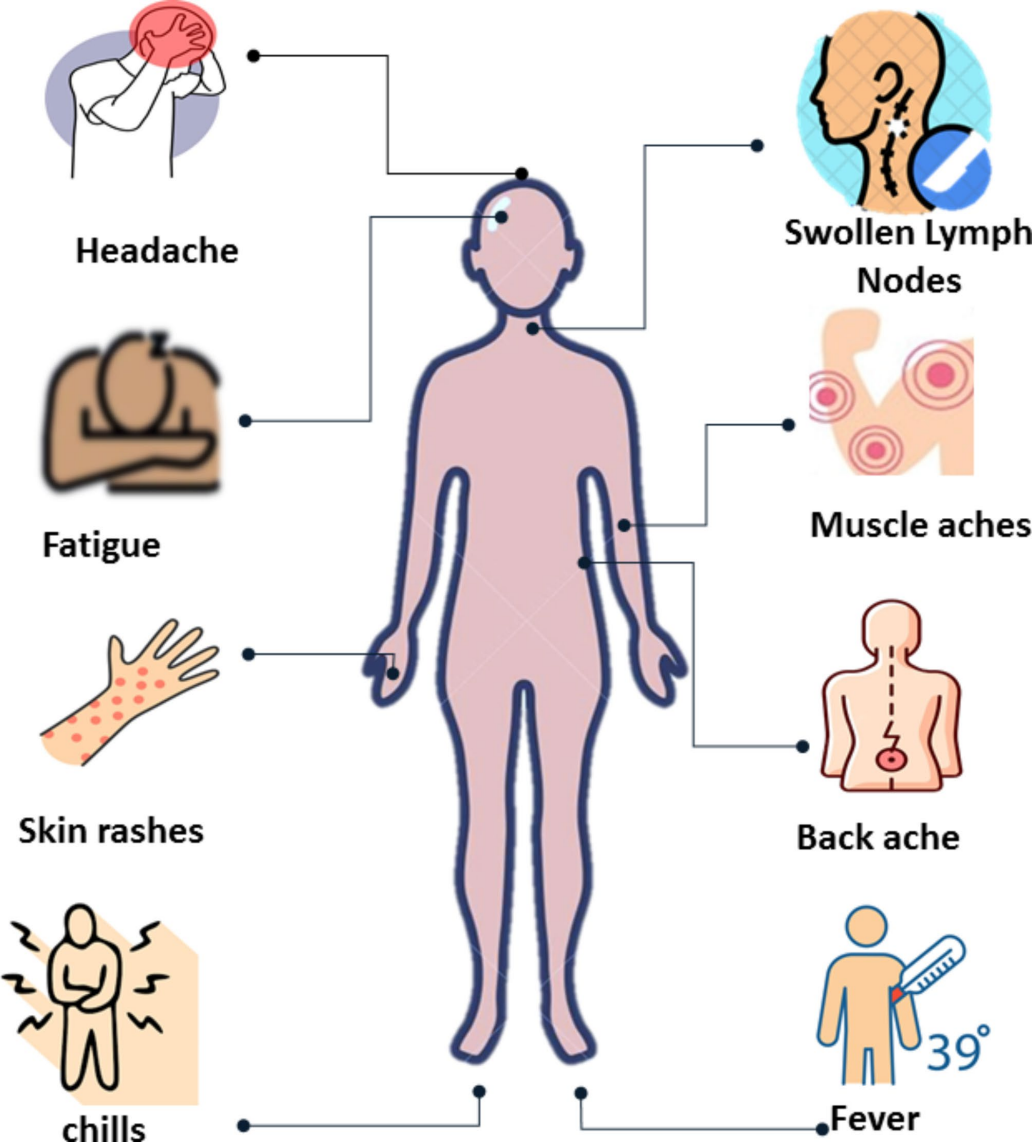
The signs and symptoms of mpox

The interval between exposure to the virus and the onset of symptoms is referred to as the incubation period, which typically ranges from seven to twenty-one days. During this period, there are no clinical signs of monkeypox, and the infected individual is not contagious [57]. The illness begins with nonspecific symptoms, such as fever, chills, headaches, muscle aches, lymph node swelling, and back pain, which typically appear one to two weeks after infection. Fever is the initial symptom to develop, occurring before the rash (Fig. 4) [58]. Lymphadenopathy is a key feature of mpox, distinguishing it from both smallpox and varicella [59]. Most patients reported having a rash (97%) was the most common, followed by chills (71%) adenopathy (71%), fever (85%), headache (65%), and myalgias (56%) [60].

The skin rash typically appears one to three days after the onset of symptoms. It initially appears as localized papular lesions, which subsequently progress to an eruption of vesiculopustular lesions [61, 62]. Depending on the route of transmission, the skin manifestations typically begin on the face and progress to the hands, legs, and feet. Anogenital lesions are the most commonly reported cutaneous manifestation [63]. Mucosal lesions can manifest as proctitis, tonsillar or oral ulcerations, or chancriform ulcers [61]. Typically, the eruption develops quickly from macules to papules, then to vesicles, pustules, and umbilicated pustules, and finally to crusts. During the eruption, mpox patients may not develop all lesions simultaneously or in the same stage [60]. Approximately two-thirds of the individuals exhibited a pleomorphic rash, while the remaining 68% had a monomorphic rash. This pleomorphic rash is a characteristic feature of mpox, as smallpox typically presents with monomorphic pustules or crusting lesions, but not both simultaneously. Additionally, the frequency of generalized centrifugal rash dispersion was higher (48.4%) compared to localized distribution (25.8%) [60]. Crust formation typically occurs 7–14 days after the onset of symptoms, and in most cases, the illness resolves within 3–4 weeks [62]. Once the patient’s crusts have completely fallen off, they are no longer considered contagious [64].

Individuals with advanced immunosuppression, young children, and pregnant women may exhibit a distinct clinical presentation of mpox [65]. Severe complications of mpox include encephalitis, myocarditis, keratitis, epiglottitis, pneumonitis, and secondary bacterial infections. While the precise risk factors for severe mpox disease sequelae are not fully understood, individuals living with HIV are generally more susceptible to these complications [61]. Overall, the death rate was 8.7%. A comprehensive review indicates that the death rate among HIV-positive individuals who contracted monkeypox ranged from 1 to 11% [64]. Many of the deaths were associated with multi-organ system failure; however, there is uncertainty regarding the extent to which mpox contributed to these fatalities [65]. Changes in the monkeypox virus are occurring rapidly and have the potential to increase its lethality [64]. Early recognition of symptoms, particularly lymphadenopathy and the progression of the rash, is crucial for accurate diagnosis and effective management. Healthcare professionals are becoming increasingly familiar with the diverse clinical presentations and treatment options for this virus [66]. Clinicians who are unfamiliar with diseases that resemble monkeypox may find it challenging to distinguish it from other viral, bacterial, or non-infectious illnesses, as many of the signs and symptoms overlap with those of other conditions. Therefore, laboratory diagnosis is particularly crucial for accurate identification [45].

## Diagnosis of MPXV

**Fig. 5:**
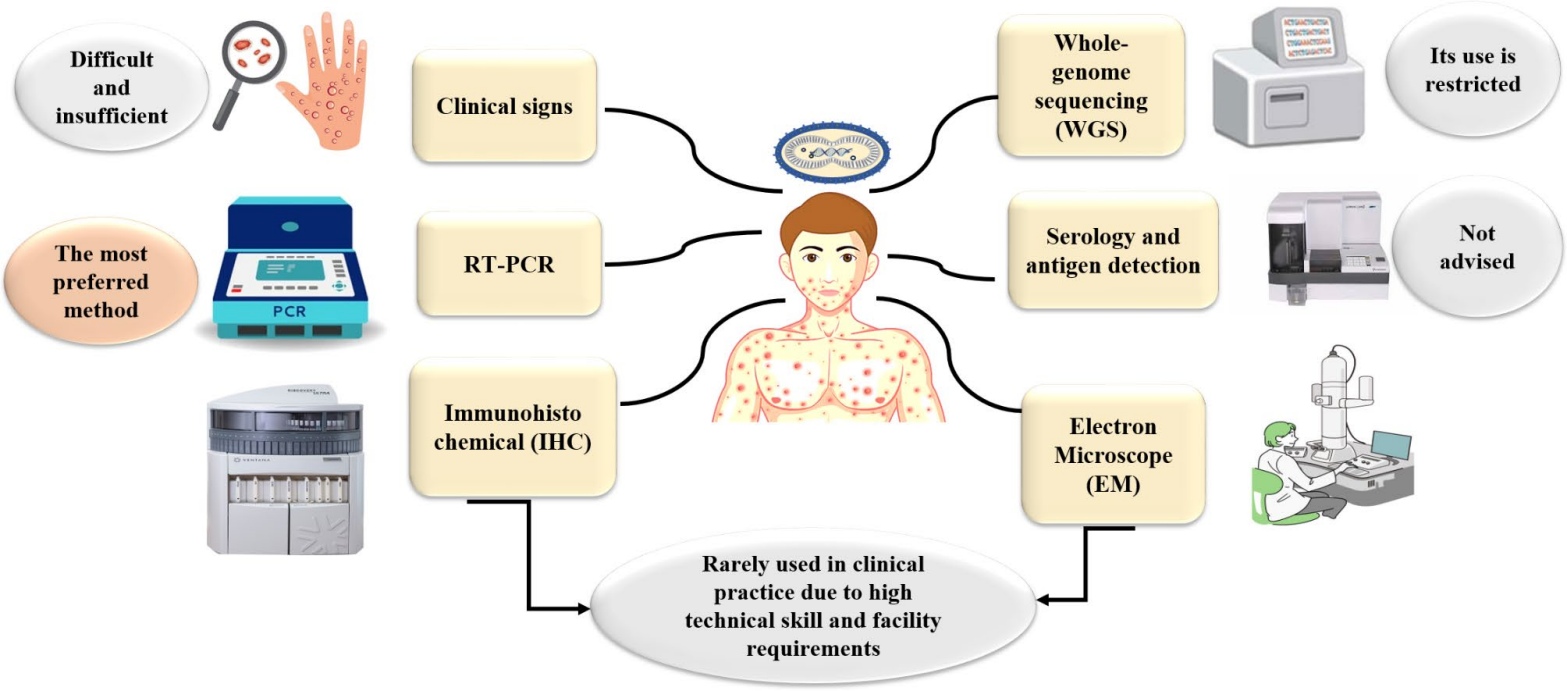
Diagnosis of mpox

If an individual displays the previously mentioned symptoms, diagnostic assessment for monkeypox should begin immediately upon clinical suspicion (Fig. 5). Notably, because of continuous human-to-human transfer, unusual patterns of transmission, and historical sexual interaction, diagnosing monkeypox during the current outbreak differs from traditional conceptions in the region of West Africa. Consequently, Even in cases where the rash is confined and not yet widespread, monkeypox might present with a STI-like rash [67]. , and should be considered when evaluating genital ulcer diseases [68]. In regions where these infections are prevalent, monkeypox is sometimes mistaken for varicella (chickenpox). Clinical suspicion and the appearance of characteristic skin and mucosal lesions are utilized to diagnose human monkeypox (MPX), which is then confirmed by molecular testing [69]. The most commonly used approach for diagnosing monkeypox is RT-PCR, which identifies viral DNA in swabs taken from ulcers, vesicle crusts, or lesions. This approach is very specific for detecting orthopoxvirus genes. Whole-genome sequencing (WGS) can be utilized for more thorough genomic analysis, although it is rarely used in routine clinical practice due to technical and resource limitations. Immunohistochemical (IHC) staining and electron microscopy (EM) are diagnostic methods utilized in specialized contexts, albeit their utilization is limited due to technical skill requirements [70]. The most valuable diagnostic samples for monkeypox testing are skin lesions, pustule and vesicle fluid, and dry crusts. Using sterile dry polyester, nylon, or Dacron swabs, at least three lesions should be swabbed to obtain two specimens. Swabs for dry lesions placed in a viral transfer medium (VTM) are acceptable for lesion crust samples [34].

### Antiviral medications and vaccines for *Orthopoxvirus* Infections

Currently, there is no specific treatment for monkeypox. However, several antiviral drugs and vaccines originally developed for smallpox may benefit individuals with monkeypox. A significant factor in the current monkeypox outbreak is the global decline in smallpox vaccination coverage. Smallpox vaccination reduces the risk of monkeypox by approximately 85% [71–74]. Because the smallpox and monkeypox viruses are similar, the vaccine protects against monkeypox as well. Decades after the discontinuation of smallpox vaccinations, the MPXV may occupy the ecological and immunological niche once held by the smallpox virus [75].

The FDA has approved several antiviral drugs and vaccines for treating monkeypox including tecovirimat [76–78], cidofovir [79–81], and brincidofovir [80–82], as well as vaccines like ACAM2000 [83], vaccinia immune globulin intravenous (VIG-IV) [84], and JYNNEOS [85]. As monkeypox cases rise globally, concerns are growing that the virus could spread widely, potentially overwhelming healthcare systems like the impact of SARS-CoV-2. JYNNEOS and ACAM2000 are two smallpox vaccinations that can cross-protect against monkeypox [86]. Research also suggests that the LC16m8 vaccine could provide long-lasting protection against MPXV [87, 88]. However, concurrent administration of antiviral medications like tecovirimat with ACAM2000 may reduce the vaccine’s efficacy [89]. Common side effects of smallpox vaccination include myalgia, lymphadenitis, malaise, lethargy, headaches, and fever [86].In rare cases, more severe side effects have been reported.

Understanding the mechanisms of action of these antiviral drugs is crucial for optimizing their use in treating monkeypox. The cidofovir, a nucleotide analog inhibits the DNA polymerase of the poxvirus by converting it into a cytidine triphosphate analog within the cell [90]. Although cidofovir is employed to treat human molluscum contagiosum poxvirus infections, its effectiveness in treating monkeypox remains uncertain [91]. Brincidofovir, a lipid compound of cidofovir, provides improved cellular absorption, a higher conversion rate to its active form, and a superior renal safety profile than cidofovir [82, 92, 93]. However, brincidofovir may elevate liver enzyme levels when administered to individuals with monkeypox [94, 95]. Another antiviral medication called tecovirimat, commercialized as TPOXX, is a small-molecule antiviral that inhibits the function of the *Orthopoxvirus* VP37 envelope-wrapping protein, which is required for the generation of egress-competent virions. Tecovirimat blocks this protein, preventing the virus from leaving infected cells and propagating throughout the host’s body [76, 96]. It is the first antiviral to be licensed by both the United States Food and Drug Administration (FDA) and the European Medicines Agency (EMA) for *orthopoxvirus* infections, including monkeypox. Tecovirimat was first licensed for smallpox in 2018, then the EMA expanded its approval to cover monkeypox in 2022, following an increase in cases in Europe. The medication was approved for treating MPXV in Europe in 2022 [97], although its combination with ACAM2000 may impair vaccine immunogenicity [89]. Clinical trials on tecovirimat have shown that it is safe and effective, with participants experiencing faster recovery in severe cases [98].

Containment methods are critical in low-resource nations with a high risk of MPXV outbreaks and limited availability to vaccines and antiviral medications. In the absence of universal vaccination, these countries can rely on early diagnosis, case isolation, and community education to prevent the virus from spreading. Furthermore, increasing diagnostic capacity and enhancing surveillance systems could assist to minimize future outbreaks and eliminate disparities in the worldwide response to MPXV.

As researchers continue to explore pharmacological interventions, interest is also growing in natural therapeutic approaches. Therapeutic approaches derived from medicinal plants are gaining traction as promising antiviral treatments. Numerous plant species have demonstrated antiviral efficacy against various viruses, and ongoing research continues to identify and characterize these natural compounds. For instance, extracts from *Alchemilla vulgaris* have shown antiviral activity against *Orthopoxvirus* [99], while *Sarracenia purpurea* has been identified as an effective inhibitor of poxvirus replication [100]. These plants may serve as promising candidates for developing novel antiviral drugs against *Orthopoxvirus* like MPV and variola.

### Social and economic impact of monkeypox resurgence

The reappearance of monkeypox presents serious public health issues, especially in areas where infectious illness cases are already disproportionately high. Monkeypox puts a strain on healthcare systems as it spreads, increasing medical expenditures, creating problems with resource allocation, and interfering with regular health services [101]. The outbreak may result in significant financial losses, especially in areas with already precarious healthcare facilities. Poverty and economic instability can be aggravated by the expenditures of treatment, hospitalization, and long-term care for affected persons, which can overwhelm local economies [102]. Furthermore, like other viral diseases, monkeypox has a social stigma that can result in discrimination and isolation from society, especially for vulnerable groups [103]. This stigma may discourage people from getting medical attention, which could lead to the infection spreading throughout the community. Misinformation and public panic about the illness may also cause people to behave differently, isolating themselves more, avoiding public places, and engaging in less economic activities. These changes in behavior may have long-term effects on economic productivity and community cohesion [85]. As previous outbreaks have demonstrated, reducing the wider effects of the monkeypox revival will require tackling the social determinants of health, raising public awareness, and guaranteeing equitable access to healthcare [75].

## Conclusion

The recent comeback of the monkeypox virus (MPXV) highlights a notable change in the virus’s epidemiological profile, moving it from predominantly being an African health concern to a global health concern. The genetic diversity and shape of the virus, particularly across Clades I and II, affect its pathogenicity and the kinetics of its transmission. The diagnostic environment has changed to match the needs of an outbreak that is fast developing, although access to modern molecular diagnostics is still difficult. To control and lessen the effects of MPXV, worldwide cooperation and ongoing surveillance are essential. Understanding the virus’s behavior and advancing diagnostic tools are critical to controlling future outbreaks and preserving public health as it spreads, especially in non-endemic areas.

## Data Availability

Data is provided within the manuscript or supplementary information files.

## Abbreviations

MPXV: Monkeypox virus
DRC: Democratic Republic of the Congo
WHO: World Health Organization
CDC: Centers for Disease Control
MSM: males who have sex with other men
USD: United states dollars
EMA: European Medicines Agency
